# Coombs-Negative Hemolytic Anemia in an Elderly COVID-19 Patient

**DOI:** 10.7759/cureus.58589

**Published:** 2024-04-19

**Authors:** Dhruvi Reddy Sanikommu, Ahmed Nadeem, Vignesh Ponnusamy

**Affiliations:** 1 Internal Medicine, New York Medical College, Landmark Medical Center, Woonsocket, USA; 2 Oncology, New York Medical College, Landmark Medical Center, Woonsocket, USA; 3 Critical Care Medicine, New York Medical College, Landmark Medical Center, Woonsocket, USA

**Keywords:** coombs negative, hematology, cd147, elderly population, non-immune hemolytic anemia, covid-19

## Abstract

COVID-19 infections are known to cause multi-organ complications. Hematological complications like autoimmune hemolytic anemia with a positive direct antiglobulin test (DAT), are commonly encountered. However, Coombs-negative hemolytic anemia is extremely rare. We report an interesting case of an elderly female with moderate-severe acute respiratory distress syndrome in the setting of COVID-19 pneumonia-causing Coombs-negative hemolytic anemia. This patient initially presented with sudden onset abdominal pain and vomiting, found to have an incarcerated inguinal hernia with small bowel obstruction (SBO) on imaging. Additionally, labs revealed positive COVID-19 antigen test and normocytic anemia. The hospital course was complicated by worsening hemolytic anemia and thrombocytopenia requiring blood products. Extensive workup for hemolysis in this patient with no prior hematological abnormalities, was negative for DAT and other conditions associated with or causative of hemolysis. At discharge, hemolytic parameters improved and on follow-up, hemoglobin returned to baseline, and repeat hemolytic parameters were normal.

This case emphasizes the importance of considering SARS-CoV-2 along with other viral infections as one of the differentials for Coombs-negative hemolytic anemia.

## Introduction

During the pandemic of the coronavirus disease 2019 (COVID-19), numerous multi-organ complications have been encountered from this infection in the clinical setting [1]. Hematological complications including autoimmune hemolytic anemia, venous thromboembolism, and disseminated intravascular coagulopathy (DIC) have been previously reported [2]. However, Coombs-negative hemolytic anemia is extremely rare with only three cases being reported so far [1,2]. This case aims to increase awareness about atypical hematological complications associated with COVID-19 infections.

This article was previously presented as an abstract at the Rhode Island regional American College of Physicians (ACP) meeting on March 15, 2023.

## Case presentation

A 70-year-old Caucasian female with a history of chronic obstructive lung disease and hypertension presented with sudden onset abdominal pain and vomiting. ​On presentation, she was hemodynamically stable. Initial laboratory values were significant for positive COVID-19 test and normocytic anemia (Hb 10.8 g/dL). Computed tomography (CT) imaging of the chest showed extensive ground glass opacities concerning COVID-19 pneumonia. CT abdomen and pelvis revealed incarcerated inguinal hernia with small bowel obstruction (SBO). 

During the hospital course, the patient underwent a robotic-assisted SBO repair. Postoperatively, she was unable to be extubated and was transferred to the ICU for hypoxic and hypercarbic respiratory failure. ​On day 2 of admission, she was found to have worsening anemia (Hb 8.7 g/dL, MCV 100 fl) with a normal platelet count (220 x 10^3^/microL) and white cell count. Hemoglobin continued to down-trend and repeat imaging of the abdomen revealed a rectus sheath hematoma without any evidence of arterial bleed. General surgery was consulted and they recommended close monitoring without any active surgical intervention. Further workup revealed positive stool for occult blood. Gastroenterology was consulted for suspected blood loss secondary to occult GI bleeding as the cause of anemia. However, endoscopy and colonoscopy did not reveal any active bleed. ​Anemia workup was significant for hemolytic anemia (elevated reticulocyte count 6.8%, corrected reticulocyte index 2.7, elevated indirect bilirubin 1.17 mg/dL, decreased haptoglobin 11 mg/dL, and increased lactate dehydrogenase 773 U/L). The direct antiglobulin test (DAT), also known as the direct Coombs test, was negative, making autoimmune hemolytic anemia less likely. Iron panel (serum iron 15 mmol/L, total iron binding capacity 320 mcg/dL, ferritin 450 ng/mL), vitamin B12 (870 pg/mL), and folate (14.2 ng/mL) were within normal limits.

On day 10 of admission, the patient developed thrombocytopenia (103 x 10^3^/microL). Drug-induced hemolysis was suspected secondary to antibiotic vancomycin or heparin products. The 4T (thrombocytopenia, timing of platelet count fall, thrombosis or other sequelae, and other causes for thrombocytopenia) score was 4 points with an intermediate probability of heparin-induced thrombocytopenia (HIT). Vancomycin and enoxaparin were discontinued until the HIT panel was negative, and anticoagulation was restarted accordingly. Due to hemolytic anemia and thrombocytopenia, thrombotic microangiopathy was on the differential. Peripheral smear did not reveal schistocytes. PLASMIC score was 5 suggestive of intermediate risk for thrombotic thrombocytopenic purpura (TTP); however, ADAMTS13 antibody activity was higher than 45%, hence ruling out TTP. Renal function remained stable without the presence of schistocytes, which is evidence against hemolytic uremic syndrome, another entity of microangiopathies. ​COVID-19 pneumonia was treated with IV steroids and Remdesivir. During the ICU stay, the patient was extubated with improvement in oxygenation.

On day 15, the patient continued to have anemia and thrombocytopenia requiring blood transfusions. The DIC panel was negative. Complement levels were normal. Repeat DAT was negative. Repeat imaging of the abdomen was done to evaluate the hematoma and check for possible splenic sequestration; however, imaging revealed a decrease in the size of the hematoma and a normal spleen. Other conditions associated with hemolysis such as paroxysmal nocturnal hemoglobinuria (PNH) and hepatitis were also ruled out with a negative PNH flow cytometry and acute hepatitis panel respectively. Bicytopenia due to hematological malignancies in this acute setting was unlikely in the absence of lymphadenopathy or blasts on peripheral smears. Bone marrow biopsy was deferred as an outpatient if no further improvement in counts was noted. After three weeks of admission and closer to discharge, hemolytic parameters improved with a hemoglobin of 8.3 g/dL, normal platelet count, indirect bilirubin, and near normal reticulocyte count. She was diagnosed to have non-immune (Coombs-negative) intravascular hemolytic anemia secondary to COVID-19 infection. On outpatient follow-up two months later, hemoglobin returned to baseline (10.3 g/dL), and repeat hemolytic parameters were normal (Table 1). 

**Table 1:** Laboratory values during admission, at discharge, and follow-up LDH: lactate dehydrogenase, HIT: heparin-induced thrombocytopenia

Lab test	Result	Reference range
Hemoglobin		
On admission	10.8 g/dL	12 – 15.5 g/dL
Day 2 of admission	8.7 g/dL	12 – 15.5 g/dL
Day 7 of admission	7.2 g/dL	12 – 15.5 g/dL
Day 15 of admission	6.1 g/dL	12 – 15.5 g/dL
At discharge (3 weeks)	8.2 g/dL	12 – 15.5 g/dL
2-month follow-up	10.5 g/dL	12 – 15.5 g/dL
WBC count	5.23 x 10^3^/microL	3.5 – 10.50 x 10^3^/microL
Platelet count		
On admission	220 x 10^3^/microL	150 – 450 x 10^3^/microL
Day 10 of admission	103 x 10^3^/microL	150 – 450 x 10^3^/microL
Day 15 of admission	78 x 10^3^/microL	150 – 450 x 10^3^/microL
At discharge	234 x 10^3^/microL	150 – 450 x 10^3^/microL
Hemolysis labs		
During admission		
-Reticulocyte count	6.80%	0 – 2%
-Total and Indirect bilirubin	2.3 mg/dL and 1.2 mg/dL	0.2 – 1.2 mg/dL and 0.0 – 0.3 mg/dL
-Haptoglobin	11 mg/dL	37 – 355 mg/dL
-LDH	773 U/L	122 – 222 U/L
2-month follow-up		
-Reticulocyte count	1.30%	0 – 2%
-Total bilirubin	0.3 mg/dL	0.2 – 1.2 mg/dL and 0.0 – 0.3 mg/dL
-Haptoglobin	195 mg/dL	37 – 355 mg/dL
-LDH	274 U/L	122 – 222 U/L
Direct Coombs test	Negative x 2	
HIT panel	PF4 antibody negative (optical density <1); Serotonin assay: negative	

## Discussion

**Figure 1:**
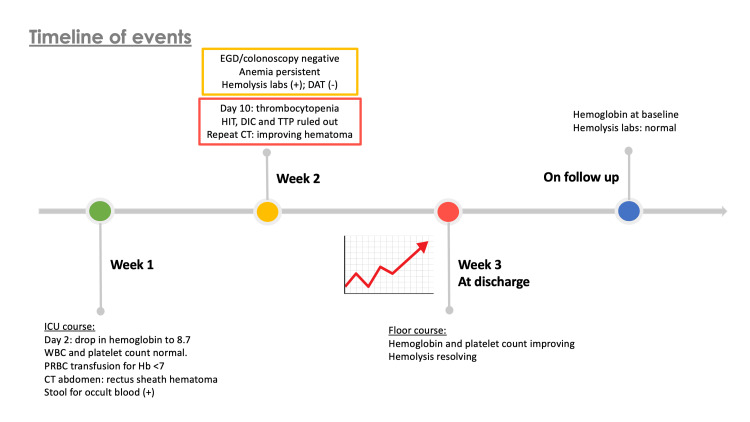
Timeline of events during the patient's admission WBC: white blood cell, PRBC: packed red blood cell, EGD: endoscopy, DAT: direct antiglobulin test, HIT: heparin-induced thrombocytopenia, DIC: disseminated intravascular coagulopathy, TTP: thrombotic thrombocytopenic purpura

Non-immune hemolytic anemia is most commonly caused by infections, medications, or hepatorenal disease [3]. Most common infections include influenza virus, cytomegalovirus, Epstein-Barr virus, Hepatitis E virus, and parvovirus B19 [2]. The workup for hemolysis in this patient with no prior hematological abnormalities, was negative for DAT and other conditions associated with, or causative of hemolysis (Figure 1), which eventually resolved on discharge. This atypical case of an elderly female with moderate acute respiratory distress syndrome was complicated with hemolytic anemia presumed to be triggered by COVID-19 infection. Autoimmune hemolytic anemia is a commonly reported complication in COVID-19 infections [4]. However, Coombs-negative hemolytic anemia is noted to be rare. As mentioned above, only three cases have been reported so far in the literature, with this case being an additional contribution. 

The pathophysiology was hypothesized to be secondary to activation of T cells by the SARS-CoV-2 virus which interacts with CD147 on the host cells, causing cytokine storm and intravascular hemolysis [2]. Another hypothesis was the possibility of low-affinity antibodies on red cell membranes or low sensitivity of the conventional tube method used for performing DAT [2,5]. There is limited literature on the exact mechanism of this viral infection causing hemolysis, and further studies are warranted to determine the same.

**Figure 2:**
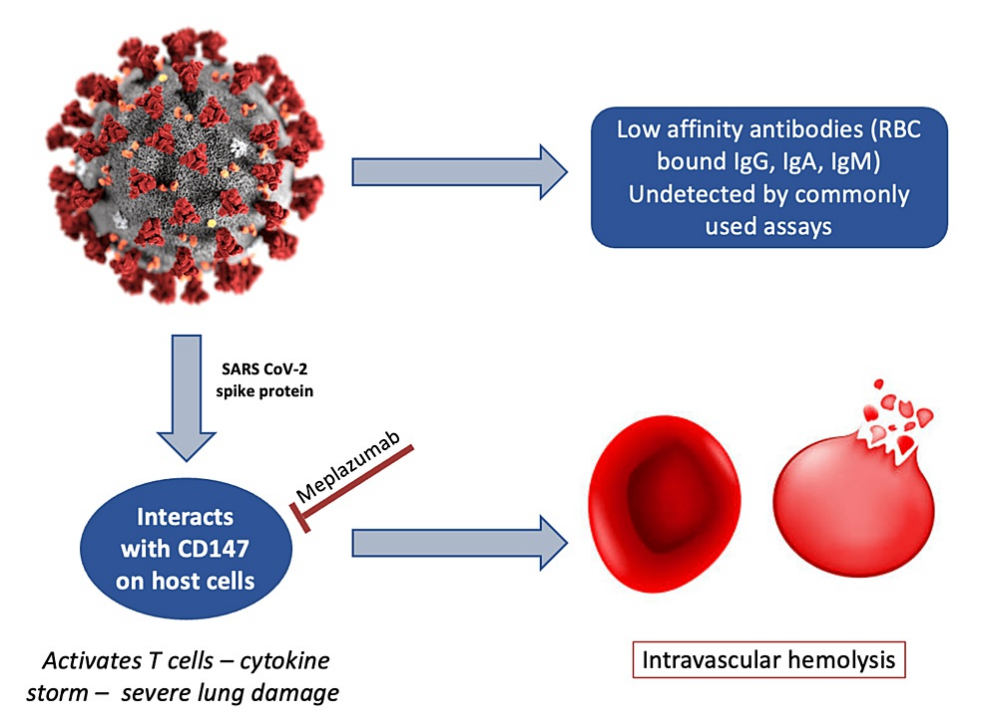
Pathophysiology of SARS-CoV2 on the red blood cells causing hemolysis The figure is originally created by the author.

There are ongoing clinical trials on the use of CD147 humanized antibody (meplazumab) in severe COVID-19 infections which could effectively inhibit viral entry and cytokine storm [6]. It would be interesting to see if this can prevent such complications in future use (Figure 2).

## Conclusions

It can be challenging for clinicians to diagnose the etiology of acute Coombs-negative hemolytic anemia in the hospital setting after extensive unremarkable workup. This case emphasizes the importance of considering SARS-CoV-2 along with other viral infections as one of the differentials for Coombs-negative hemolytic anemia.
